# The Causes and Prevention of Yellow Fever at New Orleans and Other Cities in America

**Published:** 1857-03

**Authors:** 


ART. IV.—*The Causes and Prevention of Yellow Fever at New Orleans, and other Cities, in America.* By E. H. BARTON, A.M., M.D., Chairman of the Sanitary Committee; late President of the Louisiana State Medical Society; former Professor of the Theory and Practice of Medicine and Clinical Practice in the Medical College of Louisiana, &c., &c. Third edition, with the addition of upwards of seventy pages of "Prefatory Remarks," and a Supplement. New York: T. C. Bailliere, 1857. 8vo.; pp. 282.

IGNORANCE exacts many heavy sacrifices both of person and of property, from her followers. Showing herself under a great variety of masks and ways; sometimes lackadaisical, sometimes smiling, and not seldom forward and self-sufficient, she does not frighten the multitude, like sin and death might be supposed to do, but she is in close alliance with both of them; a sleeping partner, as it were, supplying quietly, and often with a show of kind intentions, the chief materials for the others to work on and compass their ends. Neglect and exaggeration; ridicule of danger at a distance, and the basest fear when it is imminent; niggardly in preparation against coming evils; prodigal to wastefulness when they are actually felt; sneering at wisdom in the garb of science, and favoring every impudent pretender, whose garb is motley, with cap and bells,—Ignorance stands opposed to improvement in any shape. What to her is famine from neglect of agriculture, and pestilence from neglect of the precepts of hygiene! Has she not the precedent of former times and older prejudices on her side! Better, she will exclaim to the deluded multitude, that you should die in all the variety of suffering and woe, as your ancestors have died before you, good, patient, uncomplaining souls as they were, than that you should be led away by these new-fangled teachers, these visionary enthusiasts, who promise you comfort, and health, and peaceful enjoyments, which you know not of. Why go to the trouble and expense of procuring these probable benefits, even if they should prolong your lives and save your children from uncounted ills? Your lives are long enough, and why should you tax yourselves for the sake of your children? They are no better than you, and have no superior claims to escape from the filth, and the mental darkness, and the free fights, and the privilege of killing or the chance of being killed, in which you have luxuriated so long! Why rehearse this nonsense, some of our readers may ask; and yet it is by such nonsense that a community is sometimes carried away, and excuses itself for a neglect of the prevention of disease, and of demoralization and crime. Who often is so unpopular, so little thanked as he is, who sets about refuting this nonsense, and aims at the removal or even amelioration of existing evils, and the improvement of the social condition of his fellow-citizens!

We do not say that Dr. Barton, the author of the work before us, is in this category of the neglected or the illy requited; but we entertain misgivings of his not having received the full measure of reward to which he is so richly entitled, for his long, devoted, and judicious services, in bringing about an amended condition of the public hygiene of New Orleans, and with it protection from the dreadful pestilence, which, at different times, has destroyed so many of her people, and retarded so much her growth and prosperity. It may be, that, in the ardor of his belief of the necessity of reform, he does not always adhere to the most rigid methods of argument, nor express himself in a style simply didactic. He sometimes clothes aphorism in metaphor and throws spangles on science, beyond the expectations of plainer votaries. These little peculiarities, we may even magnify them into defects, do not, however, materially interfere with his array of facts, or the zeal and ability with which he brings them to bear in support of his positions. But, before we take up his line of argument and illustrations, it is proper to state the circumstances to which the present volume owes its appearance. After public attention had been thoroughly aroused to the importance of the subject by the yellow fever scourge of 1853, the Board of Health of New Orleans appointed a Sanitary Commission, consisting of the Mayor of the City, A. D. Crossman, and Drs. E.

H. Barton, A. F. Axson, S. D. McNeil, J. C. Simonds, and J. L. Riddell, whose duties were designated to be:

"1st. To inquire into the origin and mode of transmission or propagation of the late epidemic yellow fever.

"2d. To inquire into the subject of sewerage and common drains, their adaptability to the situation of our city [New Orleans], and their influence on health.

"3d. To inquire into the subject of quarantine, its uses and applications, and its influence in protecting the city from epidemic and contagious maladies; and

"4th. To make a thorough examination into the sanitary condition of the city, into all causes influencing it, in present and previous years, and to suggest the requisite sanitary measures to remove or prevent them, and into the causes of yellow fever in ports and other localities having intercourse with New Orleans."

The results of the inquiries and investigations on these several points were embodied in a volume, which was printed in 1854, under the title of "Report of the Sanitary Commission of New Orleans, on the Epidemic Yellow Fever of 1853: Published by Authority of the City Council of New Orleans." Dr. Axson reported on the first subject of inquiry; Dr. Riddell on the second; Dr. Simonds on the third; and Dr. Barton on the fourth, or on the sanitary condition of New Orleans. The present volume is a third edition of this last report, with the additions specified in the title page. Great stress is laid by the author, on the paramount importance of a thorough study of etiology; for a knowledge of causes can alone suggest the means of prevention. In its large and comprehensive sense, so as to include a connected and philosophic view of the general causes of disease, etiology, to use the language of Dr. Barton, who calls it "the basis of preventive medicine," is hardly taught in this country at all. Now, this is infinitely more important than curative medicine; preventible disease being proportioned to non-preventible disease as 8 or 10 to 1. This view may be farther enforced by a simple statement of the fact, that in 1853, thirty thousand persons, of the population of New Orleans, were attacked by yellow fever, of whom it has been estimated that upwards of eight thousand were carried off by the disease. It is easy, to a certain extent, to fill up some of the details, which must suggest themselves to every one, of the concomitant and attendant circumstances; such are, the pain and suffering, of which the bodily is not always the greatest, of the sick,—the wail of the survivors for the dead—the desolated homes—the widows, the orphans, the childless—ruined fortunes—general gloom and distress—interrupted commerce, and the loss of millions in consequence. Surely, a knowledge of the means of preventing this terrible array of ills the most afflicting to humanity, is worth obtaining; and he who offers it to us in good faith, and after much patient search and striving to discover it, deserves our thanks, although, in the end, the reality may fall somewhat short of the expectation raised.

In the "Prefatory Remarks," the author embraces the opportunity to fortify the positions taken in the Report itself, to extend its illustrations—and to give farther explanations of portions of it which have not been "so fully understood as they might have been." An opinion was expressed in the Report, that the disturbance of the original soil of a country, when the meteorological condition required to give it activity is present, has been one of the most efficient causes of every epidemic which has devastated the Southwestern parts of the United States, at least during more than half a century. To the evidence furnished by New Orleans itself, the author has added that procured from a number of towns and places in the southern region of country—some of which are introduced in this part of the work. New Orleans is represented in the Report (p. 8), "to be one of the dirtiest, and with other conjoint causes, is consequently the sickliest city in the Union, and scarcely anything has been done to remedy it." The junction of terrene and meteorological conditions, "being absolutely indispensable to the origination, transmission, and duration of yellow fever everywhere," and one of these conditions, the terrene, being under human control, and even the other, or the meteorological, susceptible of modification by the same agency, it follows that the disease in question is preventible. The gist of the Report, after a specification of the causes which, separately or combined, or in alternate action, give rise to yellow fever, consists in pointing out the means by which they are to be removed, or their virulence nullified. The medical constitution of each month of the year 1853, up to the date of the appearance of the fever, and next the epidemic constitution, are described. The influence of the latter on vegetable and animal life is also mentioned. A critical analysis of meteorological tables, containing records of temperature, rain, and humidity, justifies, in the opinion of Dr. Barton, the conclusion, that "*in every instance where the facts are known, great heat and high saturation* were the predominant conditions for the prevalence of the disease, and it was often remarked, that *the return of these conditions reproduced the fever two or three times.*"

High temperature of a certain duration, is essential to the production of yellow fever. The average temperature at midday, in the months of May and June preceding the epidemics at New Orleans, has rarely been 81·88° F., and at the same hour during the three epidemic months 83·75° F. The average temperature of the whole day for the three months was 79·51°. It rarely reaches 90° F. during the hottest part of the day. It is not meant to be asserted that the prevalence of the fever is in proportion to the temperature. Other circumstances are necessary, among which Dr. Barton places foremost high saturation, or great humidity. The conjunction of high heat and humidity is declared to be a *sine qua non.* It has been noticed in Brazil and in Demarara, that, whenever the diseases varied or changed, they were usually preceded by some variation in the climatic condition of the place. It was remarked by Dr. Blair, that extreme seasons represented by the above conditions of atmosphere, "not only modify the type of diseases but the effects of treatment; during the depths of the rainy season adynamic and congestive types are prevalent and marked; purgatives now do harm, mercury too easily salivates; thirst is diminished. There is increased action of the kidneys; there are local congestions; headaches, drowsiness, sopor, coma, watery stools." These effects have been noticed by the author, in the climate of New Orleans for many years; (p. 76.) Doctor Barton tells us, "that there is a dew-point peculiar to each of the higher classes of fever (in their aggravated or epidemic grade) is, doubtless, true from what we know of the temperature essential to their existence, and how greatly they are all increased by humidity. The dew-point of yellow fever is from 70° to 80°; the disease rarely exists long when it is under 60°. The plague has probably a dew-point of 10° less. The typhus gravior at from 35° to 45°, and the cholera in this climate varies from 48°, and sometimes much less, to 74°, and is probably less controlled by its fall than yellow fever. The sources of this great excess of humidity are, mainly, the swamps, lagoons, lakes around us, and which are also the principal causes of our fogs, imperfect drainage and want of pavements." Beyond what the author says of the dew-point in the season of the yellow fever, we need not attach importance; the data on other points are too uncertain or conjectural. *Solar Radiation* which implies the difference between the temperature in the sun's rays and in the shade, a difference more felt even than measured by the thermometer, has not, Dr. Barton conceives, heretofore attracted notice. In common phrase, the clear days of the seasons of the disease are called "yellow fever weather." "It is characterized by being very hot in the sun and cool in the shade, at the same time, on one side of the street a boiling temperature, and on the other so cool as to urge to buttoning up the coat. This uncomfortable alternation of chilliness and heat is productive not only of uncomfortable feelings, but when exaggerated, passes into disease—constitutes the first stage of yellow fever." We are told a little farther on, that the difference of temperature here described, "essentially constitutes *with other circumstances*, a sickly season;" p. 86. This physical phenomenon is described in several places, and its direct and collateral relations are descanted on by the author. In reference to the prevalent winds, we are told that during the summer they are from the east, south, southwest and southeast. "During the worst period of the epidemic, the most frequent wind was from the east. Still more remarkable was the frequency and long duration of calms," with all their injurious saturations and depression of the vital principle; p. 93. Fluctuation in the agencies both meteorological and terrene, which give rise to yellow fever, are held to be probable. The supposition, that fever arises from a deficiency of ozone, is adverted to by the author, but no experiments were made in New Orleans to test the question.

The terrene causes are next taken up, and their relatively injurious nature discussed. Under this head the author classes "all foul, filthy, organic matter passing through its decomposition, whether terrene, miasm, malaria, or what not." Some of his critical readers will ask what means this "what not;" while others may perhaps good-naturedly exclaim: Why not a what not, as well as any other hard knot! Upturning of the original soil, of which mention has been already made, is spoken of with some detail. "The first epidemic yellow fever that is recorded here is that simultaneous with excavating the earth, in digging the Canal Carondelet, and more especially its basin, in 1797." Extensive exposures of new earth in making new basins, dredging the canals, deepening the ditches, &c., preceded the epidemic of 1853. In a subsequent section, the author makes instructive albeit not novel observations on the subject of ventilation, and the value of pure air, which is contrasted with the impure air of cities; also on water being spoiled by bad air. He next inquires into miasm as a supposed product of decomposed substances, and a specific agent in the production of fever, and expresses his disbelief in such a doctrine. He thinks that, "*whatever impairs the purity of the air is pro tanto*, for the time being, the *miasm*, or rather the *malaria;*" p. 148. As we are not among the number of those who feel disposed to take up the challenge and do battle on the opposite side, we shall pass on to other matters; reserving ourselves, if need be, for a trial of strength on another occasion.

Dr. Barton affirms "as a universal fact, with the *exceptio probat regulum*, that filth of every kind, with heat and moisture, with sufficient duration, produces yellow fever;" p. 159. With a strong leaning towards the side which he advocates, we do not entertain quite such decided conviction as he does on this point. There is probably an elimination of some subtle agency, which constitutes the poison of yellow fever; but that this is the product of filth exclusively, acted on by all the admitted aerial and gaseous and electrical elements, we are not prepared to say. We believe, however, that this is the true line of etiological investigation, and that it, more than any other, promises to lead to a solution of the yet unsolved problem. We are not, therefore, of the number of those who would discourage, still less throw ridicule on the pains-taking inquirers who have noted down every change, every phenomenon presenting itself in the material world, which has been associated with the occurrence of yellow fever. It may be found eventually, that the perfect history of this disease will derive unexpected aid from the details introduced by such writers as Dr. Barton, and in still greater number and method by Dr. La Roche; just as, at the present time, we obtain a more intimate knowledge of the national character and state of society among the people of a country, in times remote from our own, by private journals and records of domestic life and of family expenses, than from all the state documents, and the acts and speeches, and battles, and conquests, which make up the received materials for general history.

Among the contributions made by Dr. Barton to the perfect history are, apropos of the parts of cities in which the yellow fever always breaks out, descriptions of the medical topographies of those in which the disease more frequently takes up its abode; such as Havana and Vera Cruz; and of others in which it has committed great ravages, such as Rio Janeiro. In this connection, he speaks of the causes which deteriorate the air of a city, and contribute to produce a slow poisoning of its inhabitants; and he designates the means of amelioration and cure, to be a better hygiene. Prefixed to the volume before us, is a "Sanitary Map of the City of New Orleans, exhibiting the location of the various nuisances, and other causes affecting the salubrity of the city, as shown in the occurrence of nearly 30,000 cases of yellow fever in the epidemic of 1853, &c., &c.," prepared by the author. In the body of the work are charts; one illustrating the influence of solar and terrestrial radiation and moisture, in the production of yellow fever in New Orleans; the other "exhibiting the annual mortality of New Orleans, per 1000 of its population for each year, together with the causes, influencing or producing it, from 1787 (with a few exceptions) to 1854." These are the work of the author. Intermediate between them, are three Meteorologic Registers of New Orleans, kept by Dr. Barton. At the end of the volume are "A table of deaths in New Orleans during the year 1853, showing for each class of diseases the total mortality, and that of each month; also the sexes and colors, with the ages and places of nativity: compiled for the Sanitary Commissioner, by D. MacGibbon, M.D.; and two meteorological tables (on one sheet) for the year 1853." They alone can fully estimate the value of the map and the charts and tables above mentioned, who should attempt to prepare anything analogous, or who are engaged in an investigation of a subject which requires for its elucidation the aid of similar illustrations. Such documents would alone entitle Dr. Barton to be exempted from any grave criticism for diffuseness in description, and the intercalation of matter not directly necessary to a narrative of the medical topography and climate, as explaining the cause of the yellow fever of New Orleans. If we might offer advice, it would be, that, in a subsequent edition of the present work, or in the preparation of one of an analogous character, on perhaps a more extended scale, he would throw into an appendix the disquisitions and details on public and private hygiene and the collateral sciences, which are now spread over his pages, and which, though valuable in themselves and germain to his theme, overlay and somewhat interfere with his free handling of what more especially belong to and constitute the essential part of his history, and the arguments included in it. At present, the work might well and quite appropriately be called A Treatise on Public Hygiene, with a more especial Application to the Causes and Prevention of Yellow Fever in New Orleans and other Cities in America. It is, we know, a somewhat unusual fault, if fault it be, in an author to give much more in his book than he promises in his title page; and if we advert to an instance of this nature in the case before us, it is that the labors of Dr. Barton may be rendered still more serviceable to his country and creditable to himself. If he were once to engage in the task of separating his present materials, and of reconstructing a new work out of them, he would require no critical friend at his elbow to point out redundancies and repetitions with some slips of logic. These he would see at once, and be the most eager to correct.

We must not conclude without some reference to the remarks of the author on the influence of social habits in the production of yellow fever. His views and experience on one of these habits, that of drinking intoxicating liquors, is stated in the following terms: "During the whole course of the sitting of the Sanitary Committee, as a court of inquiry into the causes of the epidemic, and its great mortality, the inquiry was usually made of those we examined of the influence of social habits (intemperance) upon the liability to the disease, and on its results. The answer was almost uniformly, that it not only *increased the liability to attack, but greatly lessened the chances of recovery.* This is most singularly and impressively illustrated by the record I have received from the 'Sons of Temperance,' showing that of these about five hundred remained in the city during the epidemic, of whom, only *seven* fell victims to it, the proportion being 1 in 71·42 or 1·40 per cent.; the mortality of the balance of the city, of those who remained under similar circumstances, being 1 in 15·43 or 6·48 per cent., or nearly five times as many." The most eloquent commentary on this plain narrative could scarcely add to the force of the example, which it furnishes, of the protecting power of temperance.

On the subject of the mode of transmission or extension of yellow fever, from the spot in which it first breaks out, and from the persons who are first attacked, and of the means of prophylaxis, we cannot now speak; our scant limits only having allowed of a curt notice of the general scope of the work before us. The light in which the author regards these subjects, even if he had not made a more distinct declaration of his sentiments, is readily inferred from his views of the origin of yellow fever. These are of course entirely opposed to a belief in contagion, either direct or contingent, and to restrictions on freedom of person or of commerce, based on such a belief.

